# Traffic-Related Particulate Matter and Acute Respiratory Symptoms among New York City Area Adolescents

**DOI:** 10.1289/ehp.0901499

**Published:** 2010-05-07

**Authors:** Molini M. Patel, Steven N. Chillrud, Juan C. Correa, Yair Hazi, Marian Feinberg, Deepti KC, Swati Prakash, James M. Ross, Diane Levy, Patrick L. Kinney

**Affiliations:** 1 Division of Pulmonary, Allergy, and Critical Care Medicine, Department of Medicine, College of Physicians and Surgeons, Columbia University, New York, New York, USA; 2 Lamont-Doherty Earth Observatory, Columbia University, Palisades, New York, USA; 3 Division de Salud Communitaria, Fundación Santa Fe de Bogotá, Bogotá, Colombia; 4 Department of Environmental Health Sciences, Mailman School of Public Health, Columbia University, New York, New York, USA; 5 For a Better Bronx, Bronx, New York, USA; 6 West Harlem Environmental Action, Inc., New York, New York, USA; 7 Department of Biostatistics, Mailman School of Public Health, Columbia University, New York, New York, USA

**Keywords:** asthma, black carbon, diesel traffic, respiratory symptoms, urban air pollution

## Abstract

**Background:**

Exposure to traffic-related particulate matter (PM) has been associated with adverse respiratory health outcomes in children. Diesel exhaust particles (DEPs) are a local driver of urban fine PM [aerodynamic diameter ≤ 2.5 μm (PM_2.5_)]; however, evidence linking ambient DEP exposure to acute respiratory symptoms is relatively sparse, and susceptibilities of urban and asthmatic children are inadequately characterized.

**Objectives:**

We examined associations of daily ambient black carbon (BC) concentrations, a DEP indicator, with daily respiratory symptoms among asthmatic and nonasthmatic adolescents in New York City (NYC) and a nearby suburban community.

**Methods:**

BC and PM_2.5_ were monitored continuously outside three NYC high schools and one suburban high school for 4–6 weeks, and daily symptom data were obtained from 249 subjects (57 asthmatics, 192 nonasthmatics) using diaries. Associations between pollutants and symptoms were characterized using multilevel generalized linear mixed models, and modification by urban residence and asthma status were examined.

**Results:**

Increases in BC were associated with increased wheeze, shortness of breath, and chest tightness. Multiple lags of nitrogen dioxide (NO_2_) exposure were associated with symptoms. For several symptoms, associations with BC and NO_2_ were significantly larger in magnitude among urban subjects and asthmatics compared with suburban subjects and nonasthmatics, respectively. PM_2.5_ was not consistently associated with increases in symptoms.

**Conclusions:**

Acute exposures to traffic-related pollutants such as DEPs and/or NO_2_ may contribute to increased respiratory morbidity among adolescents, and urban residents and asthmatics may be at increased risk. The findings provide support for developing additional strategies to reduce diesel emissions further, especially in populations susceptible because of environment or underlying respiratory disease.

Growing epidemiologic evidence indicates that long-term exposure to traffic-related air pollution, particularly diesel exhaust particles (DEPs), is associated with higher prevalence of asthma and chronic respiratory symptoms (Kim et al. 2004; McConnell et al. 2006). One key longitudinal study observed that exposures to traffic-related pollutants such as elemental carbon (EC), nitrogen dioxide (NO_2_), acid vapor, and fine particulate matter [PM; aerodynamic diameter ≤ 2.5 μm (PM_2.5_)] were associated with deficits in lung function growth between 10 and 18 years of age (Gauderman et al. 2004). More recently, geographic indicators such as roadway proximity have been associated with frequency of asthma exacerbation (Chang et al. 2009) and with development of allergic sensitization and chronic respiratory symptoms in birth cohort studies (Morgenstern et al. 2008; Ryan et al. 2007). Studies have also demonstrated that shorter-term increases in ambient traffic-related particles exacerbate asthma and increase airway inflammation in children with asthma (Delfino et al. 2006, 2008; Gent et al. 2009; Hirshon et al. 2008). The relative effects of ambient DEP exposure on acute respiratory morbidity in healthy children are less well understood.

Urban and suburban disparities in prevalence and acute exacerbation of asthma are well documented (Aligne et al. 2000; Bryant-Stephens 2009). Asthma hospitalization rates for children and young adults are higher in New York City (NYC) than in surrounding suburbs (New York State Department of Health 2007) and are higher in NYC communities with greater numbers of diesel emission sources, such as major trucking thoroughfares and bus depots (Lena et al. 2002; Tonne et al. 2004). Air pollution, allergens, socioeconomic status, ethnicity, health care inequalities, and psychosocial stressors have been implicated as risk factors for the disproportionate burdens of asthma prevalence and morbidity among urban children (Bryant-Stephens 2009). The interrelationships among urban residence, ambient DEP exposure, acute respiratory symptoms, and susceptibility of asthmatics are not well characterized, and assessment of health risks associated with acute DEP exposures has been impeded by the lack of exposure data at fine temporal and spatial resolution in urban and suburban communities.

In the present study, our objective was to characterize associations between daily school-based measurements of ambient black carbon (BC), an indicator of DEPs, and daily respiratory symptoms in a longitudinal study of asthmatic and nonasthmatic adolescents residing in NYC and in a nearby suburban community. Although BC is a product of multiple combustion processes (Glaser et al. 2005), in previous studies of the participating schools (Patel et al. 2009) and other NYC locations (Kinney et al. 2000; Lena et al. 2002), BC was significantly associated with volume of diesel traffic but not car traffic. Hence, BC may serve as a suitable indicator of DEPs. We also uniquely designed the study to examine whether the effects of DEP exposure on respiratory symptoms differed by asthma status and urban residence. We hypothesized that daily reports of respiratory symptoms would be positively associated with daily BC concentrations, and the estimated effect of BC exposure on symptoms would be greater among asthmatic subjects and among urban subjects.

## Materials and Methods

### Subject recruitment and data collection

We conducted an individual-level longitudinal study involving exposure and respiratory health monitoring over a 4- to 6-week period at each of four high schools located in the NYC metropolitan area. The study was approved by the Columbia University Medical Center Institutional Review Board before the start of the study. The NYC Board of Education granted permission on the condition that school identities would remain confidential.

The four participating high schools were chosen to represent a range of exposures to traffic-related air pollutants as previously described (Patel et al. 2009). In 2003, monitoring was conducted at an NYC school (U1) adjacent to a medium-size highway. In 2004, monitoring was conducted at a second NYC school (U2) adjacent to a major highway with an annual average daily traffic 2.5 times higher than that at U1 (New York State Department of Transportation 2006). The third participating NYC school in 2005 (U3) was on a two-lane street located > 500 m from a highway that prohibits commercial traffic, including most diesel vehicles. The fourth school was located in a suburban town 40 km northwest of NYC on a two-lane roadway > 3 km from a highway. The same suburban school was monitored in 2003 and 2004 concurrently with U1 and U2, respectively. Suburban school measurements in 2003 are referred to as S1, and 2004 measurements are referred to as S2.

Study participants were recruited through classroom presentations. Anonymous baseline questionnaires were initially administered to approximately 300 students at each school to obtain characteristics of the student bodies regarding demographics, smoking, housing, activity patterns, and histories of asthma and chronic respiratory symptoms. Written informed consent was obtained from participants and parents/guardians before the start of the study. Current asthma was defined as physician-diagnosed asthma plus symptoms in the previous 12 months as self-reported on baseline questionnaires, and an effort was made to recruit at least 20 asthmatics per school. As a result of enhanced recruitment strategies targeted at asthmatic subjects over the 3 years (study advertisements placed in hallways, announcements made over public address systems, and recruitment of subjects by school nurses), the number of asthmatic subjects increased each year.

Students who returned signed consent forms (44–78 per school) were given predated daily symptom diaries to record respiratory symptoms and medication use. Subjects were instructed to complete diaries every morning by recording whether they had symptoms or used medication for asthma on the previous calendar day. Symptom diaries were collected from participants on a weekly basis to minimize recall of symptoms for > 7 days. Of the students who gave informed consent, 33–70 (75–90%) per school returned diaries and were included in the study.

### Ambient pollutant data

Outdoor BC concentrations in the PM_2.5_ size range were monitored continuously at each school using a dual-beam aethalometer (model AE-21; Magee Scientific, Berkeley, CA, USA). Aethalometers were placed with air inlets outside second- or third-floor classroom windows that faced adjacent roadways. Ambient air was sampled at 4 L/min, and suspended PM_2.5_ was deposited onto quartz fiber tape. Optical density (OD) of deposited particles was measured every 5 min at a wavelength of 880 nm. BC mass concentration was quantified by multiplying differences in OD between measurements by an absorption coefficient. At the beginning and end of each monitoring year, aethalometers were operated together in the laboratory to confirm between-instrument agreement. The aethalometer had a limit of detection (LOD) of 0.16 μg/m^3^ at a 5-min resolution.

Outdoor PM_2.5_ concentrations were monitored and recorded hourly using a beta attenuation monitor (BAM; model 1020; Met One Instruments Inc., Grant Pass, OR, USA) with air inlets placed outside of the same windows as aethalometers. Air was sampled at 16.7 L/min, and PM_2.5_ mass was calculated as a function of the attenuation of beta rays (electrons) passing through particles deposited onto filter tape. The BAM had an LOD of 4.8 μg/m^3^ at a 1-hr resolution. Once per week, aethalometer and BAM flow rates were checked, and data were downloaded. Raw data were checked for aberrations and processed according to manufacturers’ instructions.

Daily ambient NO_2_ and ozone (O_3_) data were obtained from the U.S. Environmental Protection Agency’s Air Quality Systems database (U.S. Environmental Protection Agency 2007) for two community monitoring sites closest to schools. The urban site was 2.2 km from U1, 2.4 km from U2, and 9.0 km from U3. The suburban site was 40 km from the suburban school. Hourly concentrations were aggregated into daily averages for NO_2_ and maximum 8-hr moving averages for O_3_.

### Statistical analysis

We performed statistical modeling using R version 2.10.0 (R Foundation for Statistical Computing, Vienna, Austria); results with *p* < 0.05 were considered statistically significant. Observations from all schools were combined, and three-level generalized linear mixed models were fit (glmm function with logit link for binomial outcomes) to estimate the magnitude of association between pollutant concentrations and daily symptom incidence. Because of the high correlation between school-based BC and central-site NO_2_ measurements, separate models were fit with NO_2_ as the independent variable. Pollutants were matched to symptoms by calendar date. Subject and school were included in models as random intercept terms to account for correlation among outcome responses within subjects and nesting of subjects within schools, respectively. An autoregressive covariance structure was specified to account for temporal correlation among outcomes within subjects. Other covariates included an indicator variable for weekend and daily maximum 8-hr average O_3_ concentration. Season of monitoring and daily pollen concentrations measured at a National Allergy Bureau station in Brooklyn, New York, were evaluated as confounders but were not included in final models because they lacked significance and did not appreciably change pollutant effect estimates. We examined heterogeneity of effect by urban location or by current asthma status by including interaction terms for pollutant × urban school or pollutant × asthma, respectively. We performed stratified analyses if interaction terms attained a significance level of *p* < 0.10. We examined effects at multiple lags of exposure using daily averages from the same day (lag 0), previous day (lag 1), and 2-day to 5-day moving averages. Results are reported as odds ratios (ORs) and 95% confidence intervals (CIs) per interquartile range (IQR) increase in pollutant concentration using the full range of concentrations across all schools.

## Results

### Characteristics of study population

The study population consisted of 249 mostly female (71%), nonwhite (40% Hispanic, 35% black) adolescents 13–20 years of age, although distributions of several characteristics varied among individual schools (Table 1). During the study period, 46% of subjects reported any wheeze, and the median (range) percentage of subjects reporting wheeze per day was 11% (0–50%). We observed similar frequencies for shortness of breath and chest tightness (Table 1). Use of medication for asthma was less common, with 22% subjects reporting any use during the study period. Cough was the most frequently reported symptom. More than 86% subjects reported cough at least once during the study period, and the median (range) percentage of subjects reporting cough per day was 36% (15–61%).

### Trends in ambient pollutants

Daily average BC concentrations were highly variable over the study period, with the range of concentrations spanning near one order of magnitude at each school. The median (range) concentrations were, for U1, 2.4 (0.68–5.5) μg/m^3^; U2, 2.0 (0.59–4.9) μg/m^3^; U3, 1.5 (0.33–2.3) μg/m^3^; S1, 0.60 (0.11–1.8) μg/m^3^; and S2, 0.49 (0.10–2.7) μg/m^3^; (Table 2). BC showed strong, positive correlations with NO_2_ and weaker, negative correlations with O_3_ (Table 3).

## Associations between ambient pollutants and respiratory symptoms

In analyses combining data from all subjects and schools, an IQR increase in same-day average (lag 0) BC concentration (1.2 μg/m^3^) was associated with an increased OR of wheeze of 1.11 (95% CI, 1.00–1.22) (Table 4). Previous-day (lag 1) and multiday averages of BC were significantly associated with wheeze, with the largest ORs observed for 4-day (1.19) and 5-day average BC (1.22). Same-day BC was also significantly associated with shortness of breath and chest tightness. Associations of lag 1 and 2-day average BC with cough were significantly negative.

Multiple lags of NO_2_ exposure were significantly associated with wheeze and shortness of breath, and ORs increased as the number of averaging days increased (Table 4). All evaluated lags of NO_2_ were significantly associated with shortness of breath, with ORs (per 16-ppb increase in NO_2_) ranging from 1.20 for same-day NO_2_ to 1.35 for 5-day average NO_2_. Same-day and previous-day NO_2_ were not significantly associated with wheeze, whereas associations of multiday average exposures were borderline significant or significant. Similar to BC, the estimated effect of lag 1 NO_2_ on cough was significantly negative. Associations of 3-day, 4-day, and 5-day average PM_2.5_ with wheeze were significantly positive. Associations of PM_2.5_ with cough, shortness of breath, and chest tightness were consistently negative and statistically significant (Table 4).

In stratified analyses that adjusted for current asthma, the association between BC and shortness of breath was increased for urban subjects (OR = 1.26; 95% CI, 1.14–1.40) but not for suburban subjects (interaction *p* = 0.02) (Table 5). The estimated effect of BC on chest tightness was also larger and statistically significant in urban subjects compared with suburban subjects. Associations of BC with wheeze, cough, and use of medication for asthma did not significantly differ between urban and suburban groups (Table 5), so stratified analyses were not performed for these outcomes. We observed a difference between urban and suburban subjects in the association of NO_2_ with shortness of breath but not with the other outcomes examined (Table 5). As with BC, the association between NO_2_ and shortness of breath was increased among urban subjects (OR = 1.27; 95% CI, 1.14–1.42) but not among suburban subjects (OR = 0.98; 95% CI, 0.83–1.15).

Controlling for urban location, the BC × asthma interaction was statistically significant for wheeze (*p* = 0.07) and use of medication for asthma (*p* = 0.001), and the NO_2_ × asthma interaction was significant for wheeze (*p* = 0.03) and chest tightness (*p* = 0.02). In stratified analyses, effect estimates for same-day average BC and NO_2_ were larger in asthmatics (*n* = 57 from all schools) than in nonasthmatics (*n* = 192 from all schools) (Table 5). For example, the OR for the association between BC and wheeze was 1.23 (95% CI, 1.00–1.50) in asthmatics and 1.00 (95% CI, 0.89–1.13) in nonasthmatics. Similarly, the effect estimate for NO_2_ with wheeze was elevated in asthmatics (OR = 1.13; 95% CI, 0.94–1.36) but not in nonasthmatics (OR = 0.90; 95% CI, 0.79–1.02). Associations of BC with cough, shortness of breath, and chest tightness and associations of NO_2_ with cough, shortness of breath, and use of medication for asthma were comparable between asthmatics and nonasthmatics (Table 5). PM_2.5_ associations were not significantly modified by urban residence or current asthma (0.12 ≤ *p* ≤ 0.84 for interaction terms; data not shown).

## Discussion

A key aim of this study was to analyze the contribution of traffic-related PM_2.5_ to risk of acute respiratory morbidity among adolescents in the NYC metropolitan area. Further, by monitoring traffic-related particles and collecting symptom data from asthmatic and nonasthmatic subjects at urban and suburban schools, we aimed to examine whether effects vary by current asthma and by urban residence. Although current levels of ambient PM_2.5_ have been associated with respiratory morbidity in U.S. communities (Dominici et al. 2006; Gent et al. 2003; Koenig et al. 2003; O’Connor et al. 2008), uncertainty exists over which components of PM_2.5_ are responsible for observed effects. The present findings demonstrate that increases in fine BC, an indicator of DEPs, are associated with increased probability of wheeze, shortness of breath, and chest tightness. PM_2.5_ as a whole, which is a heterogeneous mix of chemical constituents from multiple sources, was not consistently associated with increases in daily symptom incidence. This study makes an important contribution to the literature by demonstrating that exposure to fine PM components emitted by diesel vehicles may confer greater risk for respiratory symptoms than does PM_2.5_ as a whole. Furthermore, residence in NYC and having asthma may independently confer susceptibility to BC-related respiratory morbidity. Ambient NO_2_, an indicator of both gasoline and diesel emissions, was also significantly associated with respiratory symptoms. Thus, all traffic may be an important source of pollutants associated with respiratory symptoms. At two study schools, volume of diesel traffic but not car traffic was associated with BC (Patel et al. 2009), which provides support for independent effects of diesel vehicle emissions in increasing adverse respiratory health effects. Nonetheless, because of the high correlation between BC and NO_2_, it is difficult to distinguish between the effects of diesel and gasoline vehicle emissions in this study.

Contrary to expectations, BC, NO_2_, and PM_2.5_ were negatively associated with cough at several lags of exposure, which could not be explained by negative correlations between cough and other symptoms or by inclusion of covariates in the model. The relatively high prevalence of cough and weak correlation with other symptoms (data not shown) suggest that other unexamined meteorologic factors or ambient exposures negatively correlated with BC, NO_2_, or PM_2.5_ may exert greater influence on incidence of cough. Negative associations of PM_2.5_ with shortness of breath and chest tightness could not be explained by negative correlations between PM_2.5_ and other pollutants in either pooled or individual school data. However, ORs for associations of same-day average PM_2.5_ with wheeze, shortness of breath, and use of medication for asthma were > 1.0, and associations of 3- to 5-day average PM_2.5_ with wheeze were positive and significant, suggesting that at certain lags PM_2.5_ exposure may increase risk of respiratory symptoms. PM_2.5_ was not measured at S1, and data were missing for most of the study period at S2 because of equipment malfunctions. Because of fewer person-days of observations and domination of PM_2.5_ analyses with observations from urban subjects, the PM_2.5_ findings may not be representative of the general population.

Our results are consistent with those from other recent studies that demonstrate adverse respiratory health effects in association with traffic-related exposures in populations not limited to children with asthma (Gauderman et al. 2004; Kim et al. 2004; Ryan et al. 2007; Salam et al. 2007). A strength of our study is its longitudinal design, involving continuous measurement of ambient BC concentrations and collection of daily respiratory symptom data from individual subjects. Each subject served as his or her own control, thus minimizing confounding from between-subject differences in time-invariant factors such as sex and ethnicity. BC concentrations spanned nearly an order of magnitude at schools and displayed acute changes day-to-day (Patel et al. 2009), which increased power to estimate their effect on changing daily incidence of symptoms. In a longitudinal study of asthmatics by Delfino et al. (2003), daily concentrations of EC, which is similar to BC, were associated with daily respiratory symptoms ascertained from diaries, and same-day averages were more strongly associated with symptoms, compared with previous-day averages. Symptoms vary day to day and thus may be a sensitive indicator of the effects of daily changes in air pollution on respiratory health. The results of both studies highlight the importance of obtaining daily data, which are subject to less recall error, compared with historical questionnaire data. With self-administered diaries, it is difficult to assess whether subjects record symptoms on a daily basis. The school-based study provided the opportunity to address this limitation by having teachers remind students to complete symptom diaries at the beginning of class each day. Another advantage of the school-based study was accessibility of study staff to subjects’ classrooms to collect diaries and reinforce the importance of completing diaries daily.

In this study, BC measurements were limited to school locations, which may not accurately represent subjects’ exposures in other locations given the high spatial heterogeneity in BC observed in NYC (Kinney et al. 2000; Lena et al. 2002) and the wide range of distances between school and home observed among subjects. School-based BC exposures were significantly associated with respiratory symptoms, and potential error in the measurement of personal exposures would be random and result in the underestimation of BC associations. Together with findings from other school-based studies (Janssen et al. 2003; Kim et al. 2004), the present findings indicate that recurrent exposures during the 6- to 7-hr school day may be independent risk factors for adverse respiratory health effects and provide rationale for policies to reduce children’s exposures to traffic-related pollutants by limiting time spent outdoors or limiting new school construction adjacent to major roadways. Alternatively, given the high urban-to-suburban correlation in daily BC at concurrently monitored study schools (Patel et al. 2009), high correlation between serial measurements of school and personal EC exposures in NYC students (Spira-Cohen et al. 2010), and high infiltration of ambient BC indoors (Sarnat et al. 2006), school-based measurements of BC may serve as reasonable estimates of personal exposure in longitudinal analyses.

Because ambient NO_2_ concentrations are spatially heterogeneous, the degree of measurement error may vary among schools because of varying distance to central site. The greatest NO_2_ measurement error potentially occurred at U3, where BC correlation with NO_2_ was the lowest. We obtained larger ORs for multiday averages of NO_2_, in which single-day measurement errors may be smoothed, suggesting that measurement error could have biased results to the null and led to underestimation of NO_2_ associations. However, multiday averages of BC also had larger estimated effects on symptoms, and for most symptoms, ORs in pooled and subgroup analyses were similarly significant for BC and NO_2_ and had CIs of similar width. Also, daily values in school-based BC and central-site NO_2_ were highly correlated at all schools but U3. These observations suggest that misclassification of NO_2_ exposure arising from use of central site measurements did not strongly affect findings.

Significant heterogeneity in BC and NO_2_ associations by urban school location, controlling for current asthma, suggests that urban residents may have increased susceptibility to the effects of traffic-related pollutants on respiratory symptoms, independent of asthma. Associations may vary between urban and suburban locations because of differences in the mix of ambient pollutants, allergens, and other environmental exposures (Matsui et al. 2008; Simons et al. 2007). Also, estimated effects may be larger in magnitude in urban subjects because of higher BC concentrations or interactions between BC and other urban risk factors for increased respiratory morbidity. In suburban-only analyses, most ORs for symptoms were close to 1.0, and the widths of CIs were not much larger than those for urban-only analyses, suggesting that lack of significance in suburban schools was not simply due to lack of power from smaller sample size. Pollutants other than BC and/or NO_2_ may have been important risk factors for respiratory symptoms in the suburban subjects during the study period.

The associations of BC and NO_2_ with respiratory symptoms were larger in asthmatics than in nonasthmatics, independent of urban location. Asthmatics may be more sensitive to BC exposures a result of increased deposition and retention of particles in airways and/or greater sensitivity to release proinflammatory cytokines (Lay et al. 2009; Quaedvlieg et al. 2006). In NYC and the United States, asthma exacerbations are a leading cause of school absenteeism and source of health care expenditures (Garg et al. 2003). Accumulating evidence of greater health risks associated with exposure to traffic-related pollutants among asthmatics has important implications for developing policies to reduce acute asthma morbidity and the associated public health consequences. The estimated effect of BC on wheeze was larger among asthmatics than among nonasthmatics and was borderline significant only in asthmatics. The estimated effect of NO_2_ on wheeze was larger among asthmatics but not significant. The CIs associated with these ORs in asthmatics were wider compared with those in pooled analyses. The relatively small sample size of asthmatics in all schools except for U3 may have resulted in reduced power to detect significant associations. Lack of sufficient power may also explain why we did not observe associations of BC with shortness of breath and chest tightness that differed significantly between asthmatics and nonasthmatics despite significant associations in pooled analyses.

The study population comprised mostly nonwhite participants, although the distributions of race and ethnicity varied among the five school cohorts. The heterogeneity in time-invariant characteristics among subjects and schools did not likely confound analysis of within-subject exposure–response associations over time, although the findings may not be generalizable to the general U.S. population or other populations that differ in distribution of demographic characteristics. Study participants differed from the schools’ student bodies according to many factors, including sex, smoking, and current asthma prevalence. The voluntary nature of participation may have also contributed to sampling bias in the study. However, we did not aim to obtain representative populations but rather to oversample asthmatics and examine differences in susceptibility between asthmatics and nonasthmatics.

DEPs are a heterogeneous mixture of EC, sulfates, nitrates, metals, trace elements, organic compounds, and particles of various sizes. In experimental studies, various components in isolation have been shown to stimulate release of proinflammatory cytokines and immunoglobulin E (Bömmel et al. 2003; Inoue et al. 2007; Kleinman et al. 2007). Although BC accounts for most DEPs by mass, BC may be serving as a surrogate for another DEP component that is a causal risk factor for respiratory morbidity. Alternatively, although BC concentrations at schools in the present study (Patel et al. 2009) were significantly associated with local diesel traffic volume, BC may be serving as a surrogate for other mobile and stationary combustion sources that emit BC (Glaser et al. 2005). Ambient concentrations of BC and PM_2.5_ were higher at urban schools than at suburban schools (Patel et al. 2009), and differences in PM_2.5_ were only partly explained by differences in BC. Therefore, other source components of PM_2.5_ are also likely to differ between urban and suburban locations and may also be important risk factors for respiratory morbidity. Identifying specific PM_2.5_ components and sources that are causally associated with adverse health effects has important implications for future air quality standards and control programs.

Exposures from other combustion sources such as domestic cooking, environmental tobacco smoke, and transportation may also be important risk factors for respiratory morbidity in this study population. In a subset of 18 U3 subjects, smoking exposures and gas stove use were stable over time within subjects and were uncorrelated with temporal trends in school-based BC concentrations. Total commute time and time spent in each mode of transport varied temporally but were uncorrelated with BC (data not shown). Based on this subset of the data, it is not likely that these factors confounded the association between BC and respiratory symptoms.

## Conclusions

The findings of the present study contribute to furthering understanding of the respiratory health effects associated with specific components of PM_2.5_ by demonstrating that increases in respiratory morbidity are associated with acute increases in BC, an indicator of DEPs, but not with PM_2.5_ as a whole. The results further suggest that asthmatics and urban populations may be more susceptible to exposure to traffic-related pollutants. Although the effect estimates for BC exposures were relatively small, they may be especially significant when considering population-level risks, given the large numbers of people exposed to high volumes of truck and bus traffic in high-density metropolitan areas and the high prevalence of asthma among their residents.

## Figures and Tables

**Table 1:** Characteristics of study population.

	School				
Characteristic	U1	U2	U3	S1	S2
Sample size	33	58	70	46	42
Age (years) [median (range)]	17 (14–18)	15 (13–18)	16 (15–20)	16 (15–20)	17 (13–19)
Sex					
Male	37	11	34	33	32
Female	63	89	66	67	68
Race/ethnicity					
White, non-Hispanic	4	0	22	21	15
Black, non-Hispanic	43	39	19	53	64
Hispanic	46	55	36	12	10
Other	7	5	23	14	10
Father’s education level					
Less than high school	46	22	19	6	13
High school graduate	23	49	57	26	42
College graduate	31	30	25	68	45
Smoking prevalence^a^	10	0	8	7	10
Asthmatics [no. (%)]	5 (16)	14 (24)	27 (39)	3 (7)	8 (21)
School asthma prevalence^b^	21	19	19	12	12
Symptom prevalence^c^ [median (range)]					
Wheeze	14 (0–23)	10 (5–19)	14 (8–23)	7 (0–14)	13 (3–21)
Cough	46 (0–61)	39 (22–61)	27 (15–39)	33 (20–46)	35 (22–44)
Shortness of breath	16 (0–33)	18 (8–28)	26 (12–43)	7 (0–20)	16 (8–31)
Chest tightness	14 (0–55)	10 (4–19)	13 (6–21)	7 (0–36)	13 (5–25)
Use of medication for asthma^c^	11 (0–27)	8 (5–13)	12 (7–16)	2 (0–8)	6 (3–13)

Data are presented as percentages unless otherwise specified. Eight subjects (1–3 per school) are missing demographic data because of uncompleted baseline questionnaires but are included in the sample sizes for each school.

a Among the subjects who returned symptom diaries, the number of subjects who reported current smoking on the baseline questionnaire divided by the number of subjects who completed the baseline questionnaire.

b The number of students who reported current asthma on the anonymous survey divided by the number of students who completed the anonymous survey at each school.

c On a given day during the study period, the number of subjects reporting presence of a symptom or use of medication divided by all subjects providing data on symptoms or medication use that day.

**Table 2:** Distributions of daily average BC and PM_2.5_ concentrations.

				Concentration (μg/m^3^)		
School	Location	Monitoring dates	Pollutant	Median	Minimum	Maximum
U1	Medium highway	4/28/2003–6/8/2003 (BC)	BC	2.4	0.68	5.5
		5/15/2003–6/8/2003 (PM_2.5_)	PM_2.5_	20.3	8.3	42.8

U2	Large highway	2/23/2004–3/28/2004	BC	2.0	0.59	4.9
			PM_2.5_	18.4	8.8	43.0

U3	Two-lane street	4/3/2005–5/8/2005	BC	1.5	0.33	2.3
			PM_2.5_	21.6	11.5	49.0

S1	Two-lane street	5/5/2003–6/16/2003	BC	0.60^a^	0.11	1.8
			PM_2.5_	N/A	N/A	N/A

S2	Two-lane street	2/23/2004–3/21/2004	BC	0.49^a^	0.10	2.7
		3/11/2004–3/21/2004	PM_2.5_	7.8	4.5	16.5

a For 2 days measures were < LOD.

**Table 3:** Spearman correlation coefficients for pollutant pairs.

School	BC–NO_2_	BC–PM_2.5_	BC–O_3_	NO_2_–PM_2.5_	NO_2_–O_3_	PM_2.5_–O_3_
U1	0.86*	0.67*	−0.15*	0.74*	0.04	0.31*
U2	0.76*	0.47*	−0.24*	0.57*	−0.61*	−0.38*
U3	0.56*	0.53*	−0.24*	0.34	−0.12	0.08*
S1	0.90*	N/A	−0.10*	N/A	−0.02	N/A
S2	0.75*	0.68*	−0.53*	0.85*	−0.54*	−0.13*

* *p* < 0.05.

**Table 4:** ORs^a^ (95% CI) for respiratory symptoms and use of medication for asthma associated with an IQR^b^ increase in pollutant concentrations at various lags of exposure.

Pollutant	Wheeze	Cough	Shortness of breath	Chest tightness	Use of medication for asthma
BC (249 subjects, 6,210 person-days)^c^					
Lag 0	1.11 (1.00–1.22)#	0.95 (0.87–1.03)	1.26 (1.14–1.38)*	1.11 (1.01–1.24)*	1.09 (0.89–1.33)
Lag 1	1.08 (1.03–1.13)*	0.91 (0.83–0.99)*	1.03 (0.93–1.13)	0.95 (0.85–1.06)	0.93 (0.75–1.14)
2-day average	1.09 (0.96–1.22)	0.90 (0.81–0.99)*	1.22 (1.08–1.36)*	1.04 (0.92–1.18)	1.02 (0.79–1.31)
3-day average	1.10 (1.03–1.18)*	0.91 (0.81–1.02)	1.13 (0.99–1.29)	0.95 (0.82–1.10)	0.91 (0.68–1.22)
4-day average	1.19 (1.06–1.33)*	0.98 (0.86–1.12)	1.11 (0.95–1.30)	0.93 (0.79–1.10)	0.82 (0.58–1.15)
5-day average	1.22 (1.08–1.38)*	1.03 (0.88–1.19)	1.15 (0.96–1.36)	0.93 (0.77–1.12)	0.79 (0.54–1.15)

NO_2_ (249 subjects, 6,555 person-days)					
Lag 0	1.03 (0.93–1.14)	0.96 (0.88–1.05)	1.20 (1.10–1.32)*	1.08 (0.97–1.20)	1.07 (0.86–1.34)
Lag 1	1.09 (0.97–1.24)	0.88 (0.80–0.98)*	1.15 (1.03–1.29)*	1.06 (0.94–1.21)	1.01 (0.77–1.32)
2-day average	1.15 (1.00–1.33)#	0.89 (0.79–1.01)	1.28 (1.12–1.46)*	1.07 (0.92–1.25)	1.06 (0.77–1.45)
3-day average	1.32 (1.11–1.56)*	0.88 (0.77–1.02)	1.32 (1.12–1.54)*	0.99 (0.83–1.18)	1.13 (0.77–1.64)
4-day average	1.57 (1.29–1.91)*	0.96 (0.82–1.13)	1.31 (1.09–1.57)*	0.91 (0.74–1.11)	1.14 (0.74–1.76)
5-day average	1.70 (1.36–2.13)*	1.05 (0.87–1.26)	1.35 (1.09–1.67)*	0.89 (0.70–1.12)	1.05 (0.64–1.73)

PM_2.5_ (200 subjects, 4,026 person-days)					
Lag 0	1.05 (0.94–1.16)	0.86 (0.78–0.94)*	1.02 (0.92–1.13)	0.87 (0.78–0.98)*	1.06 (0.85–1.33)
Lag 1	1.09 (0.98–1.21)	0.83 (0.75–0.91)*	0.86 (0.78–0.96)*	0.80 (0.71–0.90)*	0.87 (0.69–1.09)
2-day average	1.10 (0.98–1.25)	0.79 (0.70–0.88)*	0.92 (0.82–1.04)	0.79 (0.69–0.90)*	0.93 (0.71–1.21)
3-day average	1.18 (1.03–1.35)*	0.78 (0.70–0.89)*	0.86 (0.75–0.99)*	0.74 (0.64–0.86)*	0.90 (0.67–1.21)
4-day average	1.24 (1.06–1.44)*	0.78 (0.69–0.90)*	0.80 (0.69–0.93)*	0.70 (0.59–0.82)*	0.86 (0.62–1.19)
5-day average	1.23 (1.04–1.45)*	0.76 (0.65–0.88)*	0.73 (0.62–0.87)*	0.64 (0.53–0.77)*	0.78 (0.54–1.12)

a Models combine data from all schools and adjust for school, weekend, and daily maximum 8-hr average O_3_.

b IQRs are 1.2 μg/m^3^ for BC, 16 ppb for NO_2_, and 11.3 μg/m^3^ for PM_2.5_.

c Sample sizes vary among pollutant models because of differing patterns of missing pollutant measurements.

* *p* < 0.05.

# 0.05 ≤ *p* < 0.10.

**Table 5:** ORs (95% CI) for respiratory symptoms and use of medication for asthma associated with an IQR^a^ increase in same-day average pollutant concentrations, stratified by location^b^ or asthma status.^c^

Pollutant and subgroup (sample size)^d^	Wheeze for asthma	Cough	Shortness of breath	Chest tightness	Use of medication
BC					
BC × urban interaction	*p* = 0.87	*p* = 0.75	*p* = 0.02	*p* = 0.002	*p* = 0.71
Urban (161; 3,917)			1.26 (1.14–1.40)*	1.15 (1.03–1.29)*	
Suburban (88; 2,293)			0.99 (0.74–1.32)	0.79 (0.57–1.09)	
BC × asthma interaction	*p* = 0.07	*p* = 0.73	*p* = 0.93	*p* = 0.59	*p* = 0.001
Asthma (57; 1,350)	1.23 (1.00–1.50)#				1.22 (1.01–1.49)*
No asthma (192; 4,860)	1.00 (0.89–1.13)				0.81 (0.55–1.19)

NO_2_					
NO_2_ × urban interaction	*p* = 0.66	*p* = 0.20	*p* = 0.03	*p* = 0.33	*p* = 0.83
Urban			(161, 4,259)	1.27 (1.14–1.42)*	
Suburban (88; 2,296)			0.98 (0.83–1.15)		
NO_2_ × asthma interaction	*p* = 0.03	*p* = 0.24	*p* = 0.34	*p* = 0.02	*p* = 0.28
Asthma (57; 1,473)	1.13 (0.94–1.36)			1.20 (1.00–1.44)#	
No asthma (192; 5,082)	0.90 (0.79–1.02)			0.97 (0.79–1.19)	

a IQRs are 1.2 μg/m^3^ for BC and 16 ppb for NO_2_.

b Adjusted for weekend, daily maximum 8-hr average O_3_, and asthma.

c Adjusted for weekend, daily maximum 8-hr average O_3_, and urban location.

d Sample sizes refer to number of subjects and person-days of observations in each model. Person-days vary among pollutant models because of differing patterns of missing pollutant measurements.

* *p* < 0.05.

# 0.05 ≤ *p* < 0.10.
